# Surface-emitting lasers meet metasurfaces

**DOI:** 10.1038/s41377-023-01369-y

**Published:** 2024-01-31

**Authors:** Nir Shitrit

**Affiliations:** School of Electrical and Computer Engineering, Ben-Gurion University of the Negev, Be’er Sheva, 8410501 Israel

**Keywords:** Metamaterials, Lasers, LEDs and light sources

## Abstract

The integration between vertical-cavity surface-emitting lasers and metasurfaces has been demonstrated to enable on-chip high-angle illumination for total internal reflection and dark-field microscopy. Such an ultracompact combined laser–beam shaper system provides a versatile illumination module for high-contrast imaging, thus leveraging biophotonics and lab-on-a-chip devices and facilitating life-science applications.

## Abstract (web summary)

The integration between vertical-cavity surface-emitting lasers and metasurfaces has been demonstrated to enable on-chip high-angle illumination for high-contrast microscopy, providing a versatile illumination module for biophotonics and life-science applications.

## Graphical abstract

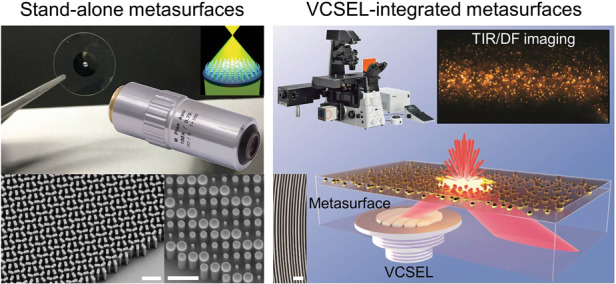

In 1801, Thomas Young performed one of the revolutionary optics experiments—the double-slit experiment—where he demonstrated a one-dimensional intensity of structured light—fringes—by the interference of a plane wave through a double slit^1^. Young’s observation of a fringe pattern proved the wave nature of light, and since then, fringe patterns have been the basis of interferometry—a fundamental technology from medical imaging to metrology and discoveries from the nature of photons to gravitational wave detection. The original high-impact double-slit experiment is a clear-cut example of the necessity of the fundamental building blocks of optical systems: light sources (in particular, lasers) and beam-shaping elements. Traditionally, lasers and beam-shaping elements are stand-alone components; however, in modern photonics, their alliance in an ultracompact on-chip platform has continuously attracted interest owing to the ever-growing demand for miniaturization while manipulating all fundamental light attributes.

Laser-wise, vertical-cavity surface-emitting lasers^2^ (VCSELs) are the unrivaled ultracompact laser sources. VCSEL is a semiconductor laser diode in which the laser cavity, including the active region, is sandwiched between bottom and top-distributed Bragg reflectors, giving rise to laser beam emission perpendicular from the top or bottom surface. VCSELs feature a small device footprint, low threshold currents, low operating voltages, and low power consumption. These characteristics, along with the high efficiency and suitability for wafer-scale production, have leveraged VCSELs in various structured light applications, such as light detection and ranging, three-dimensional imaging, facial recognition, augmented reality, and many more.

Through subwavelength structuring of materials, metamaterials^3^—exactingly designed structures—have shown exquisite control over electromagnetic properties, experimentally demonstrating phenomena not found in nature, such as a negative index of refraction and invisibility cloaking by transformation optics. Photonic metasurfaces are metamaterials with reduced dimensionality, i.e., two-dimensional ultrathin arrays of engineered subwavelength-spaced nanoscatterers that mold optical wavefronts at will by imparting phase changes along an interface^4–7^. By providing unprecedented and simultaneous control over the fundamental properties of light—phase, amplitude, and polarization—metasurfaces aim to revolutionize optical designs by realizing virtually flat, ultrathin, and lightweight optics that replace bulky optical elements. The light–scattering properties of metasurfaces can be manipulated through choices of the nanostructures’ material, size, geometry, orientation, and environment; therefore, metasurfaces open a new paradigm for flat photonics based on structured interfaces by facilitating one-to-one correspondence between information pixels and nanostructures.

Strikingly, as for the beam-shaping elements, metasurfaces are promising candidates for integration with VCSELs as metasurfaces enable custom-tailored molding of optical wavefronts by flat optics architecture. Practically, VCSELs are well-suited for monolithic integration with metasurfaces as they can be designed for a single-mode emission normal to the epitaxial structure of the chip; as such, metasurfaces can be easily integrated into the process flow for the fabrication of bottom-emitting VCSELs on transparent substrates (i.e., the lasing emission is directed to the substrate). Such an alliance of lasers—VCSELs—and beam-shaping elements—metasurfaces—opens a new paradigm for ultracompact structured light systems that feature built-in structuring of the lasing emission^8,9^ (Fig. 1). The resulting VCSEL-integrated metasurfaces benefit from a lightweight small-footprint device with a fine built-in fabrication alignment between the laser and the beam-shaping element. Successful integrations of metasurfaces with VCSELs were reported, demonstrating built-in structuring of the lasing emission directly from the VCSEL’s facet, including collimation of the diverging emission^8^, beam steering^8^, generation of Bessel^8^ and orbital angular momentum (vortex) beams^8,10^, highly uniform beam arrays by beam multiplier Dammann metasurfaces^9,11^, and circularly polarized output by chiral^12^ or geometric phase-based metasurfaces^13^.

In recent years, biophotonics has focused on label-free methods for analyzing nanoscopic objects—biological nanoparticles, biomacromolecules, drug carriers, etc.—primarily utilizing light–scattering techniques^14^. Application-wise, a recent study published in *Light: Science & Applications* by M. Juodėnas et al.^15^ takes VCSEL-integrated metasurfaces one step further, making an entry into high-contrast microscopy and biophotonics. The authors demonstrated the integration between VCSELs and metagratings that deflect the lasing emission at high angles for total internal reflection (TIR) and dark-field (DF) microscopy (Fig. 1). The functionality of the metagratings is to collimate out-of-plane the diverging lasing emission while maintaining the Gaussian divergence in-plane, giving rise to an off-axis elongated—a light sheet—illumination. This functionality (i.e., the phase profile of the metasurface) is expressed by the concept of an offset axicon. Moreover, the unique design of the metagrating nanostructures overcomes the fabrication challenges of aspect ratio-dependent etching in monolithic integration. This study opens a new paradigm for an ultracompact, versatile, and on-chip illumination module for high-contrast imaging that is cheap, efficient, and possesses a fine alignment between the laser and the beam-shaping element. The compatibility of this platform with conventional microscopy setups will leverage biophotonics and lab-on-a-chip devices, thus facilitating life-science research and applications.

**Fig. 1:**
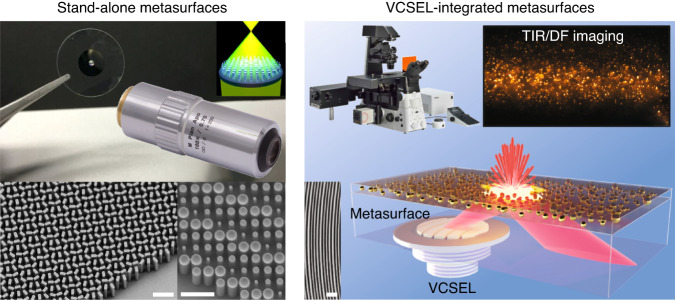
**From stand-alone metasurfaces to VCSEL-integrated metasurfaces**. Left: Illustration of a stand-alone metasurface lens (metalens) with typical polarization-dependent (bottom left) and polarization-independent (bottom right) designs; the inset shows a conventional bulky objective lens. Right: Illustration of VCSEL-integrated metasurfaces, where the curved metagratings deflect the lasing emission at high angles for high-contrast TIR and DF microscopy; the inset shows a conventional TIR microscope. All scale bars correspond to 1 μm

It is foreseeable that VCSEL-integrated metasurfaces will open a new paradigm for ultracompact on-chip illumination modules with exquisite wavefront and polarization control, giving rise to exciting opportunities for fundamental research and advanced applications. Ideally, the proposed illumination module would be combined with fluorescence imaging; however, so far, there is a lack of efficient fluorophores operating at around 1,000 nm wavelength—the operating wavelength range of GaAs-based VCSELs. While this is an active research area, VCSEL-integrated metasurfaces may further trigger the quest for suitable functional fluorophores to prove useful for fluorescence imaging. On another front, the suitability of VCSELs for wafer-scale production holds the promise to extend the scope of VCSEL-integrated metasurfaces to VCSEL arrays, which will leverage life-science applications requiring high power. Along this line, from the perspective of the gain cavity, the emergence of novel metasurface designs beyond the standard phase-only imprint will pave the way for the alliance of metasurfaces and multimode VCSELs. Moreover, advanced metasurface concepts to support ultrahigh numerical apertures will further boost the impact of VCSEL-integrated metasurfaces on high-contrast microscopy and biophotonics.

## References

[1] Young TI (1804). The Bakerian lecture. Experiments and calculations relative to physical optics. Phil. Trans. R. Soc. A.

[2] Chang-Hasnain CJ (2009). High-contrast grating VCSELs. IEEE J. Sel. Top. Quantum Electron..

[3] Kadic M (2019). 3D metamaterials. Nat. Rev. Phys..

[4] Bomzon Z (2002). Space-variant Pancharatnam–Berry phase optical elements with computer-generated subwavelength gratings. Opt. Lett..

[5] Kildishev AV, Boltasseva A, Shalaev VM (2013). Planar photonics with metasurfaces. Science.

[6] Shitrit N (2013). Spin-optical metamaterial route to spin-controlled photonics. Science.

[7] Yu N, Capasso F (2014). Flat optics with designer metasurfaces. Nat. Mater..

[8] Xie Y-Y (2020). Metasurface-integrated vertical cavity surface-emitting lasers for programmable directional lasing emissions. Nat. Nanotechnol..

[9] Shitrit, N. et al. Ultracompact structured light system of vertical-cavity surface-emitting lasers combining metagratings. *2020 Conference on Lasers and Electro-Optics*. (IEEE, San Jose, CA, USA, 2020).

[10] Sun, Y. et al. Direct generation of orbital angular momentum beams by integrating all-dielectric metasurface to vertical-cavity surface-emitting laser. *2017**Asia Communications and Photonics Conference*. (IEEE, Guangzhou, China, 2017).

[11] Shitrit N. & Chang-Hasnain, C. J. Toward 3D imaging with ultracompact structured light system of metasurface-combining VCSELs. *Imaging and Applied Optics Congress 2022*. (Optica Publishing Group, Vancouver, Canada, 2022).

[12] Jia X (2023). Metasurface reflector enables room-temperature circularly polarized emission from VCSEL. Optica.

[13] Wen D (2021). VCSELs with on‐facet metasurfaces for polarization state generation and detection. Adv. Opt. Mater..

[14] Špačková B (2022). Label-free nanofluidic scattering microscopy of size and mass of single diffusing molecules and nanoparticles. Nat. Methods.

[15] Juodėnas M (2023). High-angle deflection of metagrating-integrated laser emission for high-contrast microscopy. Light Sci. Appl..

